# Prevalence of Prediabetes and Associated Risk Factor Assessment Among Adults Attending Primary Healthcare Centers in Al Bahah, Saudi Arabia: A Cross-Sectional Study

**DOI:** 10.7759/cureus.29465

**Published:** 2022-09-22

**Authors:** Abdullah Alomari, Saad Al Hisnah

**Affiliations:** 1 Public Health Sciences, Ministry of Health Holdings, Riyadh, SAU; 2 Public Health, Ministry of Health Holdings, Al Bahah, SAU

**Keywords:** risk factors, prediabetes, prevention in primary care, primary health care centers, diabetes type 2, prediabetes screening

## Abstract

Background

Prediabetes is an asymptomatic intermediate state of hyperglycemia with a major risk of developing type 2 diabetes (T2D). It is a progressive condition; it can take a few years for patients to become diabetic from a prediabetes state.

Objectives

This study aimed to detect the prevalence rate of prediabetes among the adult population and to assess the association of the risk factors to prediabetes in both the male and female genders.

Methods

A cross-sectional study was carried out in primary healthcare centers (PHC) in the city of Al Bahah, Saudi Arabia. A total of 378 study participants of both sexes from two central PHCs were eligible to participate in the study, which was conducted from January to February 2018. Data were collected through anthropometric measurements, laboratory investigations, and a predesigned questionnaire interview to identify demographic, lifestyle, and medical variables. Two blood tests were used to diagnose prediabetes: fasting plasma glucose (FBG) and hemoglobin A1c (HbA1C).

Results

The overall prevalence of prediabetes among all participants was 20% (around 21% in males and 19% in females of all ages). There was a statistically significant association between prediabetes and obesity (central obesity), hypertension, and a family history of diabetes mellitus (FHDM).

Conclusion

The prediabetic state is highly prevalent among adults attending PHC in Al Bahah city (20%). It is associated with obesity (especially central), hypertension, and FHDM. We highly recommend that public health professionals implement strategies for effective screening, diagnosis, and management of prediabetes.

## Introduction

Diabetes mellitus (DM) is a major global health concern [1]. It is a leading cause of morbidity, mortality, and cardiovascular complications [2,3]. Prediabetes is a condition preceding diabetes wherein blood glucose is higher than normal yet below the diabetes limit [4,5]. It is typically defined as an intermediate state of hyperglycemic concentration in the blood with a high potential to progress to type 2 diabetes (T2D) [5,6]. According to the American Diabetes Association (ADA), prediabetes is diagnosed when the fasting plasma glucose (FPG) is between 100-125 mg/dl or glycated hemoglobin A1c (HbA1c) levels are between 5.7-6.4% [4-6]. The progression from prediabetes to diabetes can occur slowly over years; once the disease is established, it will be irreversible [7,8]. Therefore, awareness of the condition and immediate intervention can be indispensable to prevent or at least delay the onset of T2D [2].

Around 5-10% of all prediabetic subjects develop T2D each year in the United States [9,10]. Some studies showed a conversion to diabetes can occur within five years if prediabetes is left untreated [3]. Worldwide, residents of Asian and Middle Eastern countries are most prone to be afflicted by prediabetes and diabetes [11]. Recent data has shown that more than one-third of all adults have prediabetes in developed countries such as the United States and the United Kingdom [9,10]. In the former, approximately 86 million adults aged 20 years or above (37%) have prediabetes; approximately 90% of them are unaware that they have the condition [4,12].

Studies have confirmed a strong association between obesity and prediabetes [13,14]. Indeed, central (visceral) obesity is strongly linked to developing T2D [14]. Other risk factors include physical inactivity, hypertension, dyslipidemia (high triglycerides or low high-density lipoprotein, cholesterol), family history of diabetes, and smoking [13,14].

According to the World Health Organization (WHO), Saudi Arabia has the second-highest rate of diabetes in the Middle East and the seventh-highest globally [1]. Around 7 million Saudis (24%) are diabetic, while almost 3 million have prediabetes [15]. It is estimated that diabetes costs the Saudi government roughly 14% of its total annual health expenditure [11,15]. Moreover, there has been a significant increase in the prevalence of both conditions over the past 10 years. In fact, that increase is attributed to a variety of factors, including lifestyle changes, delayed diagnosis, a lack of awareness, and high treatment costs [9]. Consequently, there is an increasing concern over the number of subjects with prediabetes who are unaware of their condition, which might eventually progress into irreversible diabetes. Detection and activation of a treatment plan is a fundamental public health strategy to minimize and control diabetes.

Prediabetes is considered to be an asymptomatic state; the ADA thus recommends that screening should be directed at those with risk factors [6]. Adults with body mass index (BMI) ≥25 Kg/m^2^ and additional risk factors should be screened for prediabetes. If there are no risk factors, screening should not occur later than age 45. Fasting plasma glucose (FPG), two-hour plasma glucose (2h PG) after a 75g oral glucose tolerance test, and hemoglobin A1c are all validated tests to diagnose prediabetes [13].

According to the ADA, the most effective intervention measure for prediabetes is intensive lifestyle adaptation, including weight loss, physical activity, and a healthy diet. Drug-based intervention can also be taken into account to reduce the risk of progression to T2D, although it is less effective than intensive lifestyle intervention [6]. Metformin has proved effective in reducing the risk of progression to diabetes, particularly if it is administered in conjunction with extensive lifestyle adaptations. A randomized control trial of 3234 participants with elevated blood glucose revealed a reduction in the incidence of diabetes by 58% when assigned to a lifestyle modification program, while the reduction was by 31% for those assigned to metformin compared to participants receiving a placebo [16]. The severity of the disease entails further investigations and research to assess all aspects of diabetes and prediabetes in Saudi Arabia.

Our study focused on the prevalence of prediabetes among the Saudi adult population in Al Bahah City, in addition to the assessment of associated risk factors. The ADA criteria will be deployed to diagnose and assess the risk factors of prediabetes.

## Materials and methods

The study was approved by the Internal Review Board (IRB) at the College of Medicine, King Saud University. It was carried out in Al Bahah, which is located in the southern region of Saudi Arabia and has more than 80,000 inhabitants. Two central primary healthcare centers (PHC) were selected to recruit the participants. The selection of these two PHCs was based on central geographic location, daily average visitors, and availability of HbA1c blood tests. All these factors were considered to ensure that the maximum diversity of the study population was presented. The first PHC, Al Dafeer Health Center, is the largest PHC in the city, while the second is Al Bahah Health Center.

The minimum recommended sample size was 377. It was estimated by Raosoft (R) software calculator (Raosoft Inc., Seattle, Washington) [17], with a standard population size of 20,000 and a maximum prevalence rate of 50%, with an acceptable margin of error 5% and at 95% confidence level. The sample size n and margin of error E are given by x=Z(c/100)2r(100-r) n=Nx/((N-1)E2+x) E=Sqrt[(N-n)x/n(N-1)] where N is the population size, r is the fraction of responses, and Z(c/100) is the critical value for the confidence level c.

Inclusion and exclusion criteria

To be eligible for the study, participants must be between the ages of 30 and 50, Saudi, and non-diabetic. Participants who reported a history of diagnosed diabetes (HbA1c 6.5% or higher or fasting blood sugar (FBS) 126 mg/dl or higher) and pregnant females (due to gestational hyperglycemia) were excluded. In addition, participants under the age of 30 and those over the age of 50 were excluded.

A total of 378 study participants were eligible for the study from January to February 2018. The sample size was recruited equally from each of the two PHCs. We generated a specific list of participants who attended selected PHCs and met the inclusion criteria. Then, we divided the total number of required participants from each PHC by the number of days for collecting cases. A total of 10 participants from each PHC was required for each day; then, from the daily list, we used a simple random sampling technique to select 10 participants from each list.

The study subjects were subsequently approached by the principal investigator and trained nursing staff. After obtaining a signed consent form from each participant, an interview questionnaire was administered at the nursing station. A predesigned questionnaire was used to cross-reference prediabetic and normoglycemic test results with each of the associated risk factors, such as sex, age group, BMI, smoking, physical activity, waist circumference (WC), family history of diabetes, diagnosed hypertension, and family history of cardiovascular diseases (CVDs). Moreover, male and female participants were compared with regard to the associated risk factors.

An interview questionnaire was designed to collect data, including demographic and lifestyle information, medical factors, anthropometric measurements, and laboratory test results. Demographic variables included age, sex, educational level, and monthly family income. Lifestyle and medical factors included smoking status, history of hypertension, family history of DM (FHDM), family history of cardiovascular diseases, and time spent in intentional moderate physical activity per week. According to the WHO, the minimum recommended time for moderate intensity intentional physical activity is ≥ 150 minutes/week [18]. We subdivided the scale of physical activity into three categories: <60 minutes, 60-150 minutes, and ≥ 150 minutes per week.

Anthropometric parameters were measured as follows: height was measured barefoot to the nearest 0.5 cm by calibrated stadiometer, while weight was measured when participants were wearing light clothing to the nearest 0.5 kg by a calibrated scale. BMI was calculated according to WHO standards using the formula BMI=kg/m^2^, where kg is a person’s weight in kilograms and m^2^ is their height in meters squared. A BMI of 18.5 to 24.9 kg/m^2^ is classified as normal, 25-29.9 as overweight, 30-34.9 as obese class 1, and 35 and above obese class 2. Waist circumference (WC) was measured at umbilicus level by calibrated tape. For males, the first cutoff point associated with increased risk was >94 cm, compared to >80 cm for women; the second cutoff point was >102 cm for men and >88 cm for women [7]. The recommendations of the Joint National Committee were employed to measure blood pressure (BP) using a standard mercury sphygmomanometer with a cuff on the right upper arm [19]. Participants were initially seated for 10 minutes before measuring BP; three readings were taken one minute apart, then the mean of the three readings was calculated. A BP of <120/80 mm/Hg was classified to be normal; 120-139/80-89 mm/Hg was classified as prehypertensive, while 140/90 and above mm/Hg was defined as having hypertension [19]. Also, subjects reporting the current use of antihypertensive medication were classified as hypertensive.

After the collection of anthropometric measurements, the participants were sent to the lab for laboratory investigations. Two tests were considered valid for the diagnosis of prediabetes. Fasting plasma glucose (FBG) was indicated for subjects reporting fasting status eight to 10 hours before the test; otherwise, HbA1c was used. Due to a shortage of HbA1c examination kits, many of the participants reporting non-fasting were given appointments on the following day for an FBG test after fasting for eight to 10 hours. The FBG test was measured by a calibrated glucometer (ACCU-CHEK1, Model GC; Roche Holding AG, Basel, Switzerland). For HbA1c, a Quo-Test HbA1c Analyzer (EKF Diagnostics, Cardiff, United Kingdom) was used. An FBG of 100-125 mg/dl or an HbA1c value of 5.7-6.4% were classified as indicators of prediabetes [13].

Data analysis

The statistical analysis was carried out using the Statistical Package for Social Sciences (SPSS) version 20 (IBM Inc., Armonk, New York). Both descriptive and analytical statistics were conducted. Analytical methods included mean and standard deviation (SD) for quantitative variables such as age; chi-square test for comparison of sex and demographic, lifestyle, and health characteristics of participants; and the logistic regression model to calculate adjusted and unadjusted odds ratio (OR) for both sexes with its 95% confidence interval (CI). Where applicable, statistical significance was assigned at p<0.05.

## Results

A total of 378 out of 400 subjects who visited the selected PHC and were recruited to the present study consented to participate in this study, for a response rate of 94.5%. Of these, 53% were females and 47% were males. The age of participants ranged from 30-50 years, with a mean of 39 and a SD of 5.7 years. As noted in Table 1, a quarter of the participants (25.4%) were aged 30-35 years; 36% were 35-40, 21.2% were within the age of 40-45, while 17.5% of the subjects were within the range of 45-50 years.

**Table 1:** Demographic characteristics of the participants

Characteristics	Men (% of total males) (n=177)	Women (% of total females) (n=201)	Total (% of total adults) (n=378)
Age (years)			
30-35	38 (21.5%)	58 (28.9%)	96 (25.4%)
36-40	78 (44.1%)	58 (28.9%)	136 (36.0%)
41-45	34 (19.2%)	46 (22.9%)	80 (21.2%)
46-50	27 (15.3%)	39 (19.4%)	66 (17.5%)
Education level			
Primary school or less	24 (13.6%)	48 (23.9%)	72 (19.0%)
Intermediate school	22 (12.4%)	33 (16.4%)	55 (14.6%)
High school/diploma	55 (31.1%)	81 (40.3%)	136 (36.0%)
Graduate	50 (28.2%)	29 (14.4%)	79 (20.8%)
Post-graduate	26 (14.7)	10 (5.0%)	36 (9.6)
Family income per month (SAR)			
< 3000	30 (16.9%)	16 (8.0%)	46 (12.2%)
3000-5000	17 (9.6%)	52 (25.9%)	69 (18.3%)
5000-10000	61 (34.5%)	60 (29.9%)	121 (32.0%)
> 10000	69 (39.0%)	73 (36.3%)	142 (37.6%)
Work status			
Worker	155 (87.6%)	53 (26.4%)	208 (55.0%)
Non-worker	22 (12.4%)	148 (73.6%)	170 (45.0%)

Almost 10% of the participants had post-graduate qualifications; 20.8% were university graduates; 36% had a higher secondary school diploma or equivalent certification; 14.6% received intermediate school education, while 19% had completed only the primary level. Characteristics of the participants, such as family income per month and working status, are presented in Table 1.

As for lifestyle and anthropometric variables (Table 2), the prevalence of smoking was 11.6% among all participants, 22% in males, and only 2.4% in females. The majority of participants (75.1% of males and 80.1% of females) engaged in less than 60 minutes per week of intentional physical activity; around 21% of participants (22% of males and 19.9% of females) engaged in moderate physical activity, i.e., 60-150 minutes per week. Only 2.8% of males attained the recommended duration of physical activity of more than 150 minutes per week. Only 29% of participants (33% of males and 26% of females) had a BMI within the normal range (<25 kg/m^2^). Also, 31% of participants (31% in males and 32% in females) were overweight (within the BMI range 25-29.9 kg/m^2^); 30% of participants had class 1 obesity (32% in males and 30% in females), with a BMI kg/m^2^from 4430-34.9 kg/m^2^, while only 9% of total participants (4.5% of males and 12.4% of females) had class 2 obesity with a BMI of >35.

**Table 2:** Lifestyle characteristics and anthropometric measurements of the participants

Variables	Men (% of total males) (n=177)	Women (% of total females) (n=201)	Total (% of total adults) (n=378)
Smoking status			
Smoker	39 (22.0%)	5 (2.4%)	44 (11.6%)
Non-smoker	138 (78.0%)	196 (97.6.0%)	334 (88.4%)
Intentional physical activity (moderate intensity), minutes per week			
< 60	133 (75.1%)	161 (80.1%)	294 (77.8%)
60-150	39 (22.0%)	40 (19.9%)	79 (20.9%)
> 150	5 (2.8%)	0 (0%)	5 (1.3%)
Body mass index (BMI in Kg/m^2^)			
<25	58 (33%)	52 (26%)	110 (29%)
25-29.9	55 (31%)	64 (32%)	119 (31%)
30-34.9	56 (32%)	60 (30%)	116 (30%)
>35	8 (4.5%)	25 (12.4%)	33 (9 %)

Table 3 demonstrates the health characteristics of participants, showing 46.6% with a family history of diabetes (around 51% in males and 43% in females); 28.8% of participants had previously been diagnosed as hypertensive (35% among males and around 23% among females); 50.8% of participants had a family history of CVDs (around 48% among males and 54% among females).

**Table 3:** Health characteristics of the participants

	Men (% of total males) (n=177)	Women (% of total females) (n=201)	Total (% of total adults) (n=378)
Family history of diabetes			
Yes	90 (50.8%)	86 (42.8%)	176 (46.6%)
No	87 (49.2%)	115 (57.2%)	202 (53.4%)
Hypertensive (diagnosed or in treatment)			
Yes	62 (35.0%)	47 (23.4%)	109 (28.8%)
No	115 (65.0%)	154 (76.6%)	269 (71.2%)
Family history of cardiovascular diseases (CVDs)			
Yes	84 (47.5%)	108 (53.7%)	192 (50.8%)
No	93 (52.5%)	93 (46.3%)	186 (49.2%)

The overall prevalence of undiagnosed diabetes among all participants was 2% (1% among males and 1% among females), while the overall prevalence of prediabetes among all participants was 20% (21% in males and 19% in females for all ages; Table 4).

**Table 4:** Prevalence of diabetes and prediabetes among the participants by measuring either HbA1c or FBS FBS - fasting blood sugar

HbA1c				FBS				Total
Classification	Range	N	%	Classification	Range	N	%	
Normal	< 5.7 %	172	79%	Normal	< 100 mg/dl	122	77%	294 (78%)
Prediabetes	5.7–6.4 %	42	19%	Prediabetes	100–125 mg/dl	34	21%	76 (20%)
Diabetes	≥ 6.5 %	4	2%	Diabetes	≥ 126 mg/dl	4	2%	8 (2%)
Total		218	100%	Total		160	100%	378 (100%)

The effect of age and sex on the prevalence of diabetes and prediabetes is presented in Table 5. This broke down to 13% for ages 30-35, 15% for ages 36-40, 30% for (Table 5) ages 41-45, and 28% for ages 46-50 years old.

 

**Table 5:** Prevalence of prediabetes by the age group and sex of the participants

		Age (years)				Total
		30-35	35-40	40-45	45-50	
Male sex	Diabetes	0	0	2 (1%)	2 (1%)	4 (2%)
	Prediabetes	5 (3%)	10 (6%)	14 (8%)	8 (4%)	37 (21%)
	Normal	33 (19%)	60 (34%)	18 (11%)	25 (14%)	140 (77%)
	Total	38	70	34	35	177 (100%)
Female sex	Diabetes	0	0	2 (1%)	2 (1%)	4 (2%)
	Prediabetes	7 (3%)	9 (4%)	10 (5%)	13 (7%)	39 (19%)
	Normal	51 (26%)	49 (24%)	36 (18%)	26 (13%)	162 (81%)
	Total	58	58	46	39	201 (100%)
Prevalence of prediabetes among each group		12/96 (13%)	19/128 (15%)	24/80 (30%)	21/74 (28%)	76 (20%)

As shown in Table 6, the odds ratio (95% CI) of participants for anthropometric and clinical covariates associated with prediabetes, such as BMI, WC, FHDM, cardiovascular disease, and hypertension, were calculated. Both before and after OR adjustment, there were statistically significant associations between overweight and obesity with prediabetes in both males and females (p-value <0.05). Moreover, there were statistically significant associations among level 1 waist circumference, female sex, and prediabetes both before and after OR adjustment (p-value <0.05), as well as statistically significant associations among level 1 WC, male sex, and prediabetes after adjustment (p-value <0.05). There was a statistically significant association between level 2 WC and prediabetes both before and after OR adjustment in both males and females (p-value <0.05), along with a statistically significant association between diagnosed hypertension and prediabetes before and after adjustment in both males and females (p-value <0.05). We also found a statistically significant association between family history of diabetes and prediabetes after adjustment in both males and females (p-value <0.05). There were no statistically significant associations between family history of CVDs and prediabetes in either male or female subjects before and after OR adjustment, i.e., the p-value was >0.05.

**Table 6:** Adjusted and unadjusted odds ratio (OR) for anthropometric and clinical covariates associated with prediabetes ^a^ Reference is normal weight; ^b^ Reference is level 1: WC <94 cm for men, <80 cm for women; ^c^ Reference is no FHDM; ^d^ Reference is no hypertension; ^e^ Reference is no family history of CVDs WC - waist circumference, FHDM - family history of diabetes mellitus, CVD - cardiovascular disease

Risk factor	Men				Women			
	Unadjusted OR (95% CI)	p	Adjusted OR (95% CI)	p	Unadjusted OR (95% CI)	p	Adjusted OR (95% CI)	p
BMI categories								
Overweight (25-30 kg/m^2^)^a^	2.45 (1.2-6.8)	.002	1.25 (1.15-3.55)	.03	4.50 (2.25-7.9)	.016	3.78 (1.33-9.12)	.007
Obese (>30 kg/m^2^)^a^	4.484 (1.88-7.39 )	.002	1.91 (1.11-4.8)	.000	7.25 (2.5-9.77)	.005	3.91 (1.12-4.42)	.001
Risk by WC								
Moderate risk (level 1)^b^	0.87 (.39-2.7)	.821	1.49 (1.07-2.4)	.039	5.8 (1.76-13.82)	.023	2.1 (1.56-7.5)	.019
High risk (level 2)^b^	4.00 (1.74-6.73)	.025	2.73 (1.11-9.25)	.032	8.4 (2.33-15)	.006	3.69 (1.21-7.33)	.001
Family history of diabetes^ c^	1.21 (.60-3.51)	0.08	1.76 (1.2-3.6)	.010	1.09 (.87-6.42)	.095	1.70 (1.4-6.6)	.005
Diagnosed hypertension (140/90 or treatment)^d^	4.457 (1.025-6.434)	.038	2.15 (1.11-5.31)	.001	6.47 (2.22-14.9)	.047	3.43 (1.25-7.2)	.011
Family history of CVD^e^	3.925 (.262-11.03)	.239	1.47 (.24-7.74)	.997	4.67 (.73-4.3)	.132	2.8 (.78-10.1)	.113

Table 7 illustrates the adjusted and unadjusted OR for demographic and lifestyle covariates associated with prediabetes, where education level, smoking status, and level of physical activity were compared according to sex. There were no statistically significant differences between demographic and lifestyle covariates such as educational levels, smoking status, and physical activity when compared with prediabetes; the p-value was >0.05.

**Table 7:** Adjusted and unadjusted odds ratio (OR) for demographic and lifestyle covariates associated with prediabetes ^a^ Reference is primary school or less; ^b ^Reference is non-smoker; ^c ^Reference is < 60 minutes per week

Covariate	Male sex				Female sex			
	Unadjusted OR (95% CI)	p	Adjusted OR (95% CI)	p	Unadjusted OR (95% CI)	p	Adjusted OR (95% CI)	p
Education level ^a^								
Primary school or less	-	-	-	-	-	-	-	-
Intermediate school	.40 (.15-1.04)	.062	.77 (.21-2.77)	.691	.36 (.12-1.04)	.061	.431 (.09-.2.45)	.213
High school/diploma	.87 (.37-3.13)	.878	2.14 (.62-7.3)	.224	1.01 (.28-3.69)	.978	.92 (.24-3.42)	.905
Graduate	.56 (.24-4.33)	.745	1.5 (.49-3.45)	.468	.73 (.26-2.06)	.563	.67 (.23-1.92)	.465
Post-graduate	.49 (.05-4.22)	.12	1.2 (.02-4.7)	.332	.8 (.3-3.7)	.420	1.3 (.85-2.3)	.56
Smoking status ^b^								
Smoker	1.34 (1.16-2.73)	.076	.32 (.11-.91)	.063	.171 (.412-1.724)	.640	1.20 (.36-3.94)	.761
Physical activity (moderate intensity), minutes per week ^c^								
< 60	-	-	-	-	-	-	-	-
60-150	.97 (.57-1.8)	.999	1.2 (.73.3-1)	.999	1.79 (.82-3.92)	.141	2.24 (.98-5.13)	.056
> 150	1.00	1.00	2.675	1.00	0	0	0	0

## Discussion

Our study has revealed a high rate of prevalence of prediabetes among adults attending PHCs in Al Bahah. The prevalence rate was slightly higher than in other cities in the kingdom of Saudi Arabia. The overall prevalence was 20% which is relatively similar to that given in the National Survey of Health Information of Saudi Arabia, which was 17% among males and 15.5% among females [20]. This outcome was expected, as it is well known that age is the strongest predictor of diabetes and prediabetes, and our study mainly targeted participants aged 30-50 years. Our findings highlight the urgent need to implement a public health strategy for effective screening, diagnosis, and management of prediabetes in PHCs. Prediabetes is the state in which diabetes evolves; it is an asymptomatic state with a major risk of developing into type 2 diabetes. Therefore, ADA recommendations for screening should be employed for early diagnosis and intervention. Some other studies described in the literature relied on FBG measurements alone for diagnosis and thus may dramatically underestimate the true prevalence rate of prediabetes [21,22]. However, in our study, the prevalence detected by FBG was somewhat higher than (HbA1c), which may suggest an underestimation of the true prevalence for the cases addressed by FBG measurements.

Our study has also shown that the prevalence of prediabetes increased with age; the rate among people aged 40-50 (29% among both males and females) was considerably higher than those aged 30-40 years (14% among males and 7% in females). Two studies recently conducted in the kingdom supported our findings and concluded that age was the strongest predictor of DM and prediabetes [23,24].

Based on logistic regression analysis, prediabetes has a statistically significant relation with several risk factors. We found a statistically significant relationship between obesity and prediabetes. Obese male participants are twice as likely to develop prediabetes as normal participants, while obese female participants had almost four times the likelihood of developing prediabetes than normal participants. Obesity and increased body fat are associated with developing insulin resistance and T2D [20,25]. In our study, the prevalence of obesity with a BMI of >30 kg/m^2^ was 39% (36% among males, 42% among females), which is consistent with recent data about obesity in Saudi Arabia [26,27]. This modifiable risk factor should receive attention in preventive public health programs. 

In terms of abdominal obesity, which is measured by WC, there was a statistically significant relationship between high-risk level 2 in males and females, respectively, with prediabetes. That said, WC is not measured in routine clinical assessment in PHCs, although it is strongly associated with prediabetes. The lifestyle changes that are rapidly taking place in Saudi Arabia have a considerable impact on the health of society. In fact, such lifestyle transformations are believed to be responsible for the epidemic of non-communicable diseases and their complications [28].

One related risk factor to obesity is physical inactivity. We found only 3% of the male participants attaining the recommended duration of moderate-intensity intentional physical activity for ≥150 min/week, and none of the women. However, these findings regarding physical activity may be underestimated since we only estimated intentional physical activity.

Family history of diabetes was statistically associated with prediabetes; people with FHDM are at higher risk to be affected by prediabetes than those with no FHDM. Genetic predisposition to diabetes is well-documented in the literature as a non-modifiable risk factor for developing T2D. Some studies reported that people with FHDM run two to six times higher risk of developing T2D than those without such history [29,30]. It is an important public health tool to consider this factor in prevention and management strategies. Some studies showed that lifestyle intervention had a positive impact on the reduction of T2D incidence among people with FHDM with similar effectiveness as with people without FHDM [31,32].

Hypertension is widely prevalent among Saudi adult populations [31]. Prediabetic subjects with hypertension are more likely to develop T2D compared to normotensive subjects. Elevated blood pressure is associated with a metabolic syndrome that is believed to increase the risk of T2D [32]. Regarding the relation between lifestyle and demographic covariates with prediabetes, such as physical activity and smoking, no association has been established in the present study. This could be attributable to the design of the study and single-point estimation. 

We had limitations in our study. We restricted the study population to adults attending PHCs, which may affect the generalizability of the results. Moreover, we have only estimated participants’ intentional physical activity, which may underestimate overall physical activity given that we did not use the full validated global physical activity questionnaire (GPAQ) to measure physical activity. We also used European cut-off points for WC, which may not be suitable for the Saudi population.

Our strengths were that we included the most common and apparent risk factors in the assessments of predictors. To our best knowledge, this is the first study to assess prediabetes in the Al Bahah region, which is a district that desperately lacks epidemiological studies.

We highly recommend that public health professionals implement public health strategies to identify and treat prediabetes. Our recommendations are addressed to early diagnosis and immediate intervention for those most at risk of developing T2D. Obese and hypertensive populations should receive more attention in this strategy. People >40 years with no risk factors should be screened and assessed for prediabetes. Assessment of some factors, such as BMI and WC, should be included in routine medical assessments. Health education and promotion should be incorporated into prevention programs with regard to non-communicable diseases. According to the WHO, obesity is a critical yet largely neglected global issue. It can result in serious health-related events such as diabetes mellitus and threatens to overwhelm countries all over the world. With rates reaching the epidemic level in the Gulf countries, immediate action must be undertaken to control obesity. Because it is a complicated health problem, all public health agencies must cooperate to control obesity. Our recommendations include the enhancement of physical activity through the establishment of family running tracks, walking areas, cycling, walking initiatives, and school-based physical activities, in addition to strict control of diet-related factors. Finally, the many programs at the community level to control diets, such as health school campaigns and taxation of junk food, should be activated and evaluated.

## Conclusions

In conclusion, the prediabetes state is highly prevalent among adults attending PHCs in Al Bahah (20%). It is associated with obesity (especially central), hypertension, and FHDM. Our findings are in accordance with many regional studies. The fact that lifestyle, socioeconomic and dietary factors are almost identical across the Gulf region can account for this similarity. We suggest that further studies be conducted to assess the magnitude of prediabetes at the community level.
